# Acoustic simulation of cochlear implant sound to approximate the perceptual experience of electric hearing

**DOI:** 10.1038/s41598-025-25711-z

**Published:** 2025-11-07

**Authors:** Anna C. Kopsch, Stefan K. Plontke, Torsten Rahne

**Affiliations:** Department of Otorhinolaryngology, Head and Neck Surgery, Martin Luther University Halle-Wittenberg, University Medicine Halle, Ernst-Grube-Str. 40, 06120 Halle (Saale), Germany

**Keywords:** Cochlear implant, Single-sided deafness, Sound samples, Cochlear implant simulation, Outcomes research, Biological physics

## Abstract

The electrical signal processing of cochlear implants (CIs) is thought to partially explain the unnatural and unfamiliar sound impressions in recipients. To date, there are no accurate German-language sound samples available that represent how CI users perceive their CIs. The primary aim of this study was to create German-language sound samples for the sound of Cochlear Nucleus implants (Cochlear Ltd., Sydney, Australia). Furthermore, we investigated whether the simulation parameters best matched for one sentence were also accurate approximations of the CI sound when applied to two further sentences spoken by the same male speaker. Fifteen patients with single-sided deafness who had at least two years of experience with their CI were included in this study. The participants rated ten simulations based on the similarity to the sound perceived with their devices. The simulation with the best similarity score served as the starting point for optimization using a software sound-tool. On average, a score of 9.7 ± 0.5 on a scale of 1 (no similarity) to 10 (signals are identical) was achieved for the optimized simulations. Most often, a low-pass filter or a comb filter was required to optimize the simulation. The sound samples optimized for each study participant are provided in the Supplemental Material. When the parameters of the optimized simulation were applied to two further sentences (variable in content and phonemes), the similarity scores were significantly worse (mean sentence 2: 8.4 ± 1.5, *Z* = 3.015, *p* = 0.003, mean sentence 3: 8.9 ± 1.3, sign test: *Z* = 2.268, *p* = 0.048). However, when the similarity scores were considered individually for each participant, there were 4 (sentence 2) or 7 (sentence 3) participants, respectively, for whom the speech material had no influence on the similarity score.

**Supplementary Information:**

The online version contains supplementary material available at 10.1038/s41598-025-25711-z.

## Introduction

The cochlear implant (CI) is the most used neural prosthesis in the world^1^. It enables deaf and hearing-impaired people to hear by electrically stimulating the auditory nerve. Current multichannel CIs use between 12 and 24 electrodes distributed in an array along the inside of the cochlea. An audio processor is used to record acoustic signals and process them into an electrical stimulation pattern (coding strategy). In commonly used coding strategies, the audio processor digitizes the acoustic signal and divides it into a discrete number of frequency bands using a filter bank. Depending on the CI manufacturer, the filter bank is implemented either as a band-pass- or fast Fourier transform (FFT) filter bank^2^. Frequencies between approximately 200 and 8000 Hz are typically processed^2^. The coding strategy determines which information from the frequency bands is used for stimulation. The signal processing and application of the coding strategy to the acoustic signal leads to a loss of information. In addition, the spectral resolution of CIs is limited by the number and spatial separation of electrical contacts, the spread of the electric field within the cochlea and the health of the biological tissue^3–8^. The electrical signal processing of CI systems and the limited spectral resolution are thought to partially explain the unnatural and unfamiliar sound experience for patients whose implants were activated for the first time^9,10^.

Therefore, rehabilitation after audio processor activation is essential to assisting CI users in learning to interpret and understand new sensory impressions^11^, with auditory–verbal therapy being one of the commonly used approaches. Hearing rehabilitation is achieved through the interplay between the technical device programming of the CI and intensive auditory training with speech therapists within the first two years after activation, which is the standard in the follow-up therapy in Germany^12^. The CI recipients performance and thus the sound of a CI significantly changes during this time^11,13^.

Sound experience with a CI is highly relevant for the counseling and education of potential CI candidates and their social contacts and might contribute to the optimization of CI technology and CI coding strategies in the future^9,10,14,15^. Good preoperative consultation and preparation should lead to better acceptance of unnatural sound impressions during implant activation and follow-up therapy. In particular, psychosocial support from the patient’s social network can influence the success of rehabilitation. Therefore, it is important for them to have a good understanding of the patient’s situation^16,17^. Presenting sound samples of “CI simulations” to CI candidates with residual hearing and to their social contacts could support this process in most cases.

Although CIs have been implanted since 1961^18^, patients with single-sided deafness (SSD) only started receiving CIs in 2008^19^, which is why it was difficult to investigate the sound of a CI precisely. Since those patients can compare acoustic hearing with electrical stimulation (e.g.,^10,14,20,26–28,43,47^), the sound of a CI has become a primary focus of current research.

Vocoder simulations are widely studied and available on the internet as “CI simulations”^10,14,20–23^ . Vocoders are electronic devices that come from military telephone technology. They use similar signal processing of speech signals to CIs and thus mirror the information content that CIs transmit^2^. In SSD patients, Dorman et al., however, reported that vocoders do not adequately reflect the sound impressions of CIs^14^. Using various coding strategies, they reported a rather poor median similarity score for sine-vocoded signals of 2.9 on a scale of 0 (no similarity) to 10 (signals are identical) in a cohort of patients with devices from different manufacturers^14^.

The poor similarity between vocoder simulations and CI sounds may be due to the individuality of CI sounds and the ability of the human brain to reorganize in response to new stimuli (neuronal plasticity, ^44^). Thus, further signal processing techniques are necessary to mimic the CI sound with an acoustic simulation. For example, frequency shifting and high-pass filtering can mimic a frequency-to-place mismatch (difference between the frequency range of an electrode contact and the physiological correct place of processing of that frequency range). Furthermore, simple filters (band-pass, high-pass, low-pass or comb filters) can mimic the filter bank used in CIs.

Dorman et al. attempted for the first time to approximate the CI impressions of 14 SSD patients with further signal processing techniques such as those described above^15^. A speech signal was presented to the CI ear, and a modified version was presented to the contralateral ear with normal hearing. The speech signal was modified using a software sound-tool by applying various filters, vocoders, and other spectral modifications. The study participants were asked how the signal would need to be modified to match the sound of the CI. According to the study participants’ responses, the speech signal was adjusted until no better approximation to the CI sound was possible. On average, a similarity of 8.8 could be achieved on a scale of 1 (no similarity) and 10 (signals are identical). The cohort also showed large variability in the sound impressions of their CIs. The main predictors of CI-mediated speech were band-pass or low-pass filtering and spectral peak smearing^15^.

CI performance, and specifically the sound of a CI, depends on different factors. Buchman et al. and Büchner et al. independently reported that the length of the electrode array influences the development of speech recognition during follow-up therapy^13,24^. Blamey et al. reported that CI performance is also affected by the age at onset of hearing loss, age at implantation, duration of deafness, etiology, and duration of CI experience^11^. In a small case series of six bilateral CI users implanted with a Cochlear Nucleus device (CI24R/RE Contour Advance or Straight electrode, Sydney, Australia) in one ear and a MED-EL device on the contralateral ear, Harris et al. reported subjectively better sound quality in the MED-EL device^25^.

The design outlined above has been the subject of multiple investigations by Dorman et al.^9,14,15,26–28^. The authors investigated, e.g., CI sounds in CI users implanted with devices from different manufacturers (Advanced Bionics versus MED-EL,^27,28^). They reported differences in sound perception between CI manufacturers, particularly in pitch, and attributed these differences to different electrode array lengths^27,28^. In addition to the electrode array length, the electrode arrays of different manufacturers also differ in their intracochlear position (lateral wall, perimodiolar, or mid-scalar) and the number of electrode contacts^29^. In addition, audio processors from different manufacturers use different speech coding strategies, which results in differences in stimulation patterns^30^. It is possible that sound impressions may thus also depend on hardware, software (e.g., used by different companies), and other factors influencing CI performance. To date, no CI simulations for Cochlear Nucleus devices with large similarity to the CI sound exist.

The sound experience with a CI is highly relevant for the counseling and education of potential CI candidates and their social contacts. To date, no German-language CI simulations exist that accurately reflect CI sounds. As every language has its own phonemes, intonation and rhythm, it is important to create CI simulations in different languages to realistically assess the sound perceptions of CI users.

One aim of this work was to develop CI simulations that best represent the sound of a Nucleus CI for a German three-word phrase (“sentence”) for CI users who had completed follow-up therapy. A multidimensional approach was used to identify sound modification techniques that lead to the best similarity score across different speech signals.

## Materials and methods

### Study design and ethics

A monocentric, prospective, non-interventional, exploratory study was performed at the Department of Otorhinolaryngology, Head and Neck Surgery at the University Hospital Halle (Saale), Germany, from August 2022 to July 2023. All participants included in this study received all necessary information and consented to participate in the study. The study protocol was approved by the ethical review board of the University Medicine Halle (approval number: 2022–048) in accordance with the Declaration of Helsinki.

### Participants

Cochlear implant users with a normal hearing (age-appropriate) contralateral ear were included. The participants used a Nucleus device (CIx12, CIx22, CIx32, or CI24(RE), Cochlear Ltd., Sydney, Australia). The contralateral pure-tone hearing thresholds for air-conduction, averaged over 0.5, 1, 2, and 4 kHz (4PTA), were not worse than the age- and sex-related 95th percentiles according to ISO 7029^31^. All participants completed their follow-up therapy and had at least two years of CI experience. Further inclusion criteria were a postlingually acquired CI indication, a word recognition score in the German Freiburger monosyllables test at a sound pressure level of 65 dB of more than 50% with the CI and being a German native speaker. The exclusion criteria were having fewer than 20 functioning electrode contacts, electrode array malpositions (tip-foldover or incomplete electrode array insertion), cochlear implantation after surgical removal of an inner ear schwannoma, fibrosing or ossifying labyrinthitis. Postoperative X-rays from the patient’s records were used to assess the electrode array positions. Participants’ characteristics were documented.

A sample size calculation was performed with the assumption of a standard deviation of the primary endpoint (similarity score) of 1.5, three dropouts and the requirement to find the mean value with a 95% confidence interval and a precision of 2 (length of the confidence interval). This resulted in a sample size of 15 participants.

### Procedures

The participant and the experimenter sat together in a quiet room. All study measurements were performed by the same experimenter. The validated speech material of the Oldenburg children’s sentence test (OlKiSa, 1st test list: the first 10 sentences) spoken by one male speaker served as the audio signal^32^. The OlKiSa was used because it is a well-established speech test in Germany, spoken by a trained speaker. In the OlKiSa, each sentence is built as follows: number—adjective—object. One original (unprocessed) sentence was presented to the CI by a wireless TV streamer (Cochlear Ltd., Sydney, Australia). The participants used their everyday CI settings during the study experiments. The simulated (modified) audio signal was presented via an insert earphone (ER-3C, Etymotic Research Inc., Elk Grove Village, USA) to the contralateral ear. The sound volume of both signals was individually set to a subjectively comfortable level for each participant. The experimenter heard the simulated audio signal by a separate insert earphone (ER-3C, Etymotic Research Inc., Elk Grove Village, USA).

#### Screening experiment

The original audio signal was modified according to specific parameter sets (Table 1) based on those in^10,15^. The modified audio signals are referred to as “simulations” throughout this article. By applying the 10 parameter sets to 10 different sentences of the OlKiSa^32^, 10 test blocks of 10 simulations each (each block was based on a different sentence) were defined. After the original audio signal was presented to the CI ear, one of the 10 simulations of the test block was presented to the ear with normal hearing. After each presentation of a pair of sentences (original and simulated), the participants were asked to rate the similarity of the two on a subjective, discrete scale from 1 (no similarity) to 10 (signals are identical) in steps of 0.5. The participant was allowed to hear the pair of sentences several times.

**Table 1:**
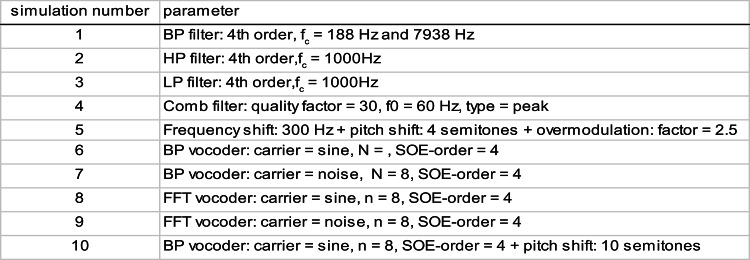
Simulation parameter sets. *BP* band-pass, *LP* low-pass, *HP* high-pass, *FFT* fast Fourier transform, *SOE* spread of excitation, *f*_*c*_ critical filter frequency, *f*_*0*_ frequency range of comb filter.

After completion of a test block, the next test block was performed as described above. In total, a series of 100 stimuli were presented to each participant. The simulations in each test block were presented in random order. The sentences were also provided to the participants in written form so that they could read them. In this way, we could ensure that the participants did not have to concentrate on understanding the words.

Before starting the study tasks, the participants completed a training task. It consisted of a short version of the screening experiment. The aim of the training task was to familiarize the participants with the simulations and handle the similarity score scale.

#### Optimization experiment

For each participant, the parameter set with the largest median similarity score over all sentences was determined. This parameter set was used as a starting point for the following optimization process. The parameter set was applied to the sentence (“drei rote Schuhe” [”three red shoes”],^32^). First, the participant heard the simulation with the normal hearing ear, and then the original signal was presented to the CI ear. The following optimization process was based on the approach of Dorman et al.^15^. The participants were asked how the simulation should be changed to make it more similar to the CI sound. For this purpose, the participant was provided with a list of adjectives adapted from Dorman et al.^15^ to help describe the sound. The participant was allowed to hear the CI simulation and the unprocessed signal presented to the CI ear several times. The simulation was then modified according to the participants’ response and was played back to the participant again. For example, if a muffled CI sound was described, a low-pass filter was used in the simulation. The cut-off frequency and the order of the filter were then adjusted until the desired level of muffling was achieved. The parameter set identified during the screening experiment served as initial starting point, but could be further modified or deactivated during the optimization procedure if necessary. This procedure was repeated until no further improvement of the simulation was achieved. Once the final simulation was found, the participant rated the similarity of the simulation to the sound of the CI ear on a subjective scale from 1 to 10 (see above). Finally, the participants were asked, one last time, how the simulation could be improved to assess the limitations of the software sound-tool.

#### Speech material experiment

The determined parameters of the optimized simulation were applied to two further sentences of the OlKiSa (“vier nasse Autos”, [”four wet cars”] and “neun weiße Tassen” [”nine white cups”]). The participants were again asked to rate the similarity of the simulation.

### Spectral signal processing

Signal processing was performed using Python (version 3.9.7, ^45^) in the integrated development environment Spyder (version 5.1.5,^46^). A graphical user interface (GUI) was developed to ease and accelerate the process. The Python library SciPy was used to read and write audio wav files in the following processing steps^33^.

#### Low-, high-, band-pass and comb filter

Comb, low-pass, high-pass, and band-pass filters were implemented. The GUI offered the option to choose the order (first to 10th) and the cut-off frequency (between 0 and 8 kHz in steps of 0.1 kHz). This corresponds to the frequency range in which most of the sound energy of the audio signal used is located and matches the full bandwidth of the filter-bank of the Advanced Combination Encoder (ACE) strategy (16 Hz,^34^) in the Custom Sound Pro (Cochlear Ltd., Sydney, Australia) programming software^35^. A low-pass filter generates a damped/muffled sound. The sound of a high-pass filtered signal is less voluminous and far away. The sound of the band-pass filtered signal is a combination of the low- and high-pass filters.

The comb filter has a magnitude response consisting of peaks with a fixed spacing of f_0_. The spacing was determined by the condition that the sampling frequency f_s_ = 44.1 kHz must be divisible by f_0_ and that f_0_ must meet the Nyquist theorem. For this reason, the GUI gave the option to set f_0_ between 1 and 14.7 kHz. A second optional parameter is the quality factor, which describes the ratio of f_0_ and the bandwidth at − 3 dB^33^. To generate a variety of sounds, the quality factor was set between 5 and 300 in the GUI. The use of a comb filter is equivalent to the audio effect named flanging^15^. The sound of the comb filter ranges from echo-like to reverberant to metallic to sharp and wheezy for varying quality factors and f_0_. The comb filter mimics the use of a filter-bank, similar to CI signal processing.

#### Pitch shift

The pitch of the audio signal was shifted using the function pitch_shift available in the Python wrapper PyRubberband^36^. The graphical user interface allows the user to shift the pitch between 0 and 15 semitones in both directions (in steps of 1 semitone, toward larger or lower frequencies).

#### Frequency shift

The spectrum of the audio signal was shifted up or down by a fixed frequency. It was set between − 30 and + 800 Hz in steps of 5 Hz. By shifting the spectrum toward larger or smaller frequencies, the harmonic relations were destroyed.

The frequency and pitch shift functions were implemented to generate a high-pitched sound in a harmonic and disharmonic variant. It simulates the spectral mismatch of CIs that have no full cochlear coverage^15^.

#### Overmodulation/clipping

The audio signal intensity was multiplied by a factor and cut off. In the GUI, the factor was set between 1.0 (no clipping) and 20.0 (very strong clipping, sounds such as a weak amplitude modulated sizzling) in steps of 0.1.

#### Fast fourier transform (FFT) vocoder

A vocoder was implemented in our GUI, which divides the audio signal into 22 frequency bands using an FFT filter bank^37^. To mimic CI signal processing, the sampling rate and the upper and lower cut-off frequencies were set according to the specifications of the ACE strategy (sampling rate: 16 kHz,^34,35^). The n bands with the largest amplitudes were selected. In the GUI n can be set between 1 and 22 (n-of-m)^34^. The remaining channels were set equal to zero. The FFT-coefficients of the n channels were used to modulate a carrier by multiplying the coefficients and a carrier^37^. The GUI also provides the option to choose between a noise- or a sine-carrier. For noise-carrier, a filter-bank was applied to broadband Gaussian noise to implement a narrowband noise bank. Finally, the modulated carriers were summed, and a vocoded signal was generated.

To simulate a wide or narrow spread of excitation (SOE), the slope (order) of the band-pass filter for the narrow-noise bands according to^38^ was variable. In the GUI, the SOE was set to narrow, moderate or flat (i.e., 4th, 2nd and 1st order of the filter).

#### Band-pass (BP) vocoder

A vocoder was implemented, which divides the audio signal into n frequency bands using a band-pass filter-bank^38^. The filter-bank and carrier specifications were the same as those described for the FFT vocoder. In the GUI, n was set between 1 and 22. As with the FFT vocoder, the simulation of the SOE was varied via the slope of the noise band carrier.

#### Other functions

An echo function and a reverberation function were implemented for the optimization experiment. These values were not required by any participant for optimization. For this reason, further details on the implementation of these functions are omitted.

### Data analysis

The similarity scores and the number of parameters used were descriptively analyzed by calculating the measure of central tendency (mean and median) and dispersion. Statistical analysis of the data was performed using the Statistical Package for Social Sciences (SPSS) software^39^. The data were tested for normality by using the Shapiro–Wilk-Test. A repeated measures analysis of variance (ANOVA) was performed to identify the effects of the sentences and simulations parameter sets on the similarity scores in the screening experiment. To account for violations of the sphericity assumptions, the Greenhouse-Geißer correction was applied. Variance homogeneity was visually inspected and confirmed. Bonferroni correction was used for post hoc tests. The best similarity scores from the screening experiment for sentence 1 were identified for each participant. A Wilcoxon signed-rank test was performed to determine if there was a difference in similarity scores between these similarity scores from the screening experiment and the optimization experiment. The Friedman test and the sign test were used to compare the similarity scores between the sentences from the speech material experiment. The significance level was set to 0.05 and was corrected by the Bonferroni correction for post hoc comparisons.

## Results

Fifteen patients (7 male and 8 female) participated in this study. For all participants, complete measurements were obtained. The mean age of the analyzed participants was (62 ± 18) years. The mean CI experience was (5 ± 2) years. In one participant (#210), two medial electrodes were deactivated. In all the other participants, all the electrodes were activated. The participants’ characteristics are summarized in Table 2 and are sorted by the CI experience of the participants. All participants’ regular CI settings were based on the ACE signal processing strategy.

**Table 2:**
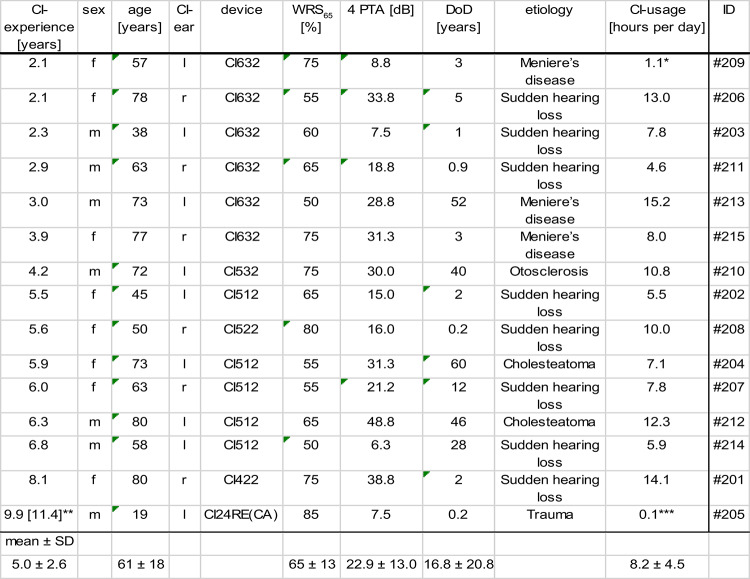
Characteristics of the participants sorted by CI experience. *f* female, *m* male, *l* left, *r* right, *WRS*_*65*_ Word recognition score measured with the German Freiburger monosyllables test at 65 dB SPL in quiet, *4 PTA* four frequency pure tone average at 0.5, 1, 2 and 4 kHz of the contralateral (not implanted) ear, *DoD* duration of deafness, *ID* identification number, *SD* standard deviation. *Short duration of usage due to comorbidity. **Reimplantation 9.9 years before participating in this study. ***Short duration of usage, as the participant subjectively does not rely on CI.

### Screening experiment

**Fig. 1:**
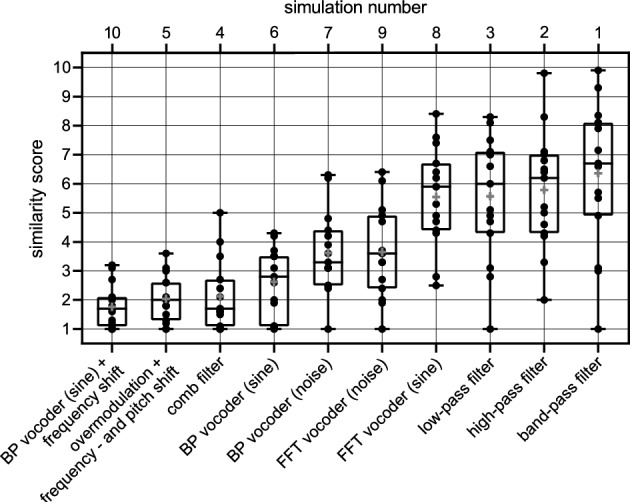
Similarity scores (1: no similarity to the CI sound, 10: signals are identical) for ten specific simulation parameter sets (simulation numbers). For each participant, the scores for ten sentences to which each of the parameter sets were applied were averaged and plotted as a point. The boxes show the lower and upper quartiles (25 and 75th percentiles) of the mean similarity scores. The horizontal lines mark the median, and the crosses mark the means of the similarity scores across all the study participants. Whiskers indicate the minimum and maximum mean similarity scores. In the diagram, the simulations were sorted according to the mean scores. In the experiments, the simulations were presented in random order. For details on the parameter settings, see Table 1. *BP* band-pass, *FFT* fast Fourier transform.

Figure 1 shows the similarity scores of the ten simulation parameter sets defined above. One data point shows the mean similarity score across the ten sentences for each participant and parameter set.

The repeated measures ANOVA indicated a significant effect of simulation parameter sets on similarity scores (*F*(2.27, 31.77) = 21.12, *p* < 0.001). In contrast, no significant main effect was found for the sentences (*F*(1.99, 27.85) = 1.85, *p* = 0.17). In the median, the band-pass filtered sound had the highest similarity to the CI sound (median similarity score of simulation 1: 6.7). The FFT sinusoidal vocoder achieved a similarity score comparable to the low-, high-, or band-pass filter scores (median similarity score ~ 6). Strong signal changes such as the BP vocoder, frequency and pitch shift combined with overmodulation, or the comb filter with strong filter characteristics led to a low similarity score in the median (e.g., median of simulation 10: 1.7). The post-hoc tests showed that parameter sets 1, 2, 3 and 8 yielded larger similarity scores than sets 4, 5, 6, and 10 (*p* < 0.001).

Both FFT vocoders were rated with a higher similarity score (median of simulation 8: 5.9) than the BP vocoder (median of simulation 7: 3.3). Among the FFT vocoders, the sine-carrier yielded a higher similarity score (median of simulation 8: 5.9) than did the noise-carrier (median similarity score of simulation 9: 3.6).

### Optimization experiment

**Fig. 2:**
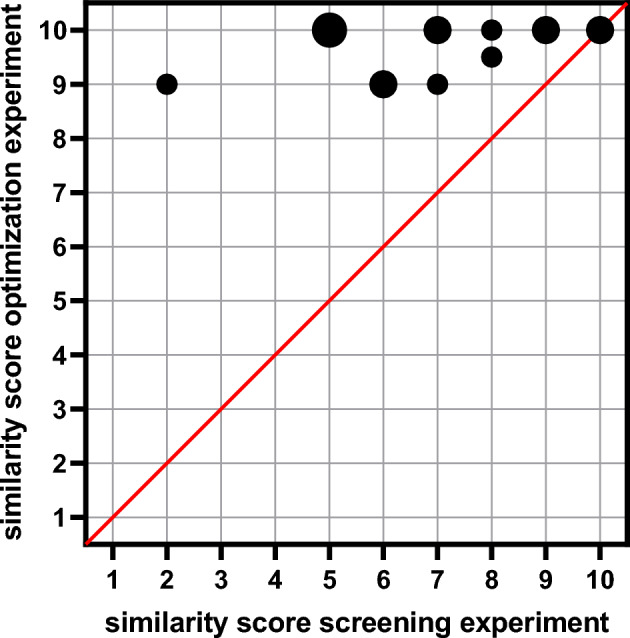
Similarity scores (1: no similarity to the CI sound, 10: signals are identical) from the optimization experiment and best similarity scores from the screening experiment for sentence 1. Each data point represents the threshold of a single participant (n = 15). Overlapping data points are larger. The line of identity is illustrated in red.

The sound files of the resulting optimized simulations for all participants and the original speech signals^32^ are included in the Supplemental Material. On average, a similarity score of 9.7 ± 0.5 (median: 10.0) was obtained. Similarity scores between 9.0 and 10.0 were reported. Ten study participants (67%) gave the best possible similarity score of 10.0. Figure 2 shows similarity scores from the optimization experiment and similarity scores from the screening experiment for sentence 1. All similarity scores are on or above the line of identity. The Wilcoxon signed-rank test revealed significantly larger median similarity scores in the optimization experiment than in the screening experiment (*Z* = 3.195, *p* < 0.001).

**Fig. 3:**
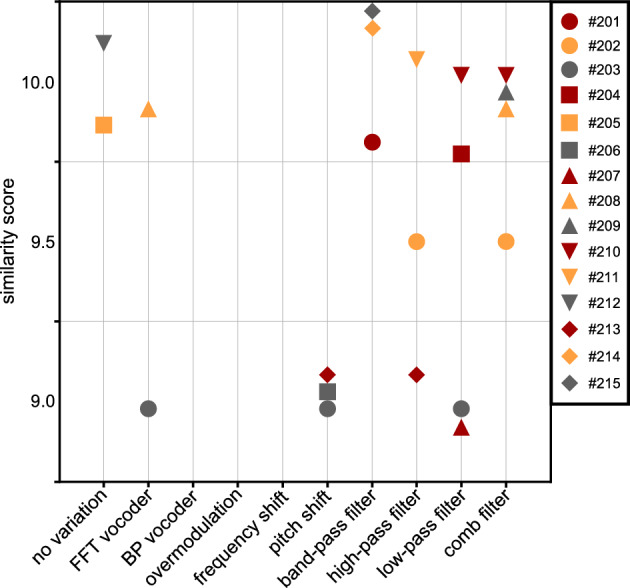
The best similarity scores achieved (1: no similarity to the CI sound, 10: signals are identical) as a function of the parameters required to optimize the simulations to the sound of a CI for each study participant. Each colorized symbol represents one study participant. If a symbol appears more than once, the optimal simulation was a combination of several parameters applied to the speech signal. By counting the number of occurrences of the symbols, the number of needed changes is assessed. For more details on the parameter settings, see Table 3. *BP* band-pass, *FFT* fast Fourier transform.

The modifications required to achieve the similarity scores shown in Fig. 2 are plotted for each study participant in (Fig. 3). On average, 1.4 ± 0.7 modifications were needed to best match the simulations to the CI sounds. Two study participants (#205 and #212) required no modification of the speech signal to best match their CI sounds. The largest number of modifications to best match the CI sound of a participant was 3 (#203). The BP vocoder, frequency shift, and overmodulation did not need to be used for any of the participants. A low-pass filter and a comb filter were used most often (4 study participants each).

When the simulations are sorted according to the participants’ CI experiences, an experienced listener can perceive an adaptation of speech sound impressions toward the sound of a normal hearing ear. Two of the participants (#203, #206) who participated in the study shortly after finishing the follow-up therapy (CI experience < 2.4 years) required strong changes in the speech signal, for example, the FFT vocoder and pitch shift. The four study participants who had the longest CI experience in this study (CI experience > 6 years) required no or very small changes (band-pass filter with a wide frequency band, Fig. 3 and Table 3, sound samples #212, #214, #201, and #205 in the Supplemental Material). The participants who required no or very small changes in CI simulations had between 50% und 85% speech recognition in the speech recognition test (Table 2). One of the participants for whom no changes to the speech signal were necessarily had a usage time of 0.1 h per day (Table 2, #205). This participant (#205) received the CI after a short duration of deafness (2 months), 11.4 years before participating in this study. A reimplantation was performed 9.9 years before participating in the study because of an implant defect. The participant showed intensive all-day usage during the first years after implantation but very limited usage (only 0.1 h per day) in recent years.

**Table 3:**
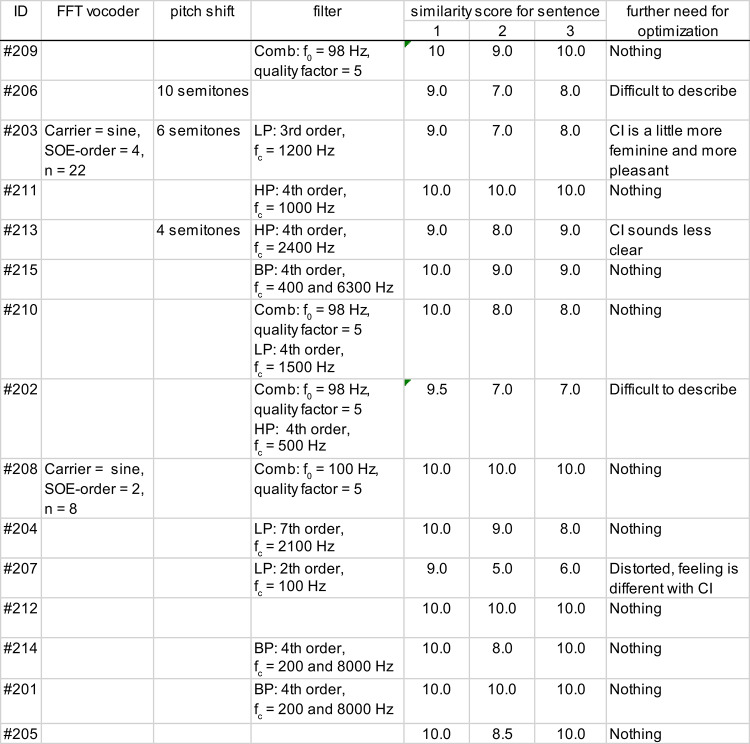
Parameters required to optimize the simulations to the sound of a CI for each participant and their similarity scores for three sentences. *BP* band-pass, *LP* low-pass, *HP* high-pass, *FFT* fast Fourier transform, *SOE* spread of excitation, *f*_*c*_ critical filter frequency, *f*_*0*_ frequency range of comb filter. 1: “drei rote Schuhe” [”three red shoes”], 2: “vier nasse Autos” [”four wet cars”], 3: “neun weiße Tassen” [“nine white cups”]. Last column: the participants’ responses to the final question after the optimization procedure about how the simulation would need to be modified to achieve higher similarity.

Table 3 summarizes the parameters of the simulations for each study participant. The participants’ responses to the final question after the optimization procedure (how the simulation would need to be modified to achieve higher similarity) are given in the last column of (Table 3). The 10 participants who rated the optimized simulation with a similarity score of 10.0 indicated that no further modification of the simulations were needed. Two other participants could not describe what needed to be changed. The remaining three participants described the sound perceived with their CI as more feminine, more pleasant, less clear, or more distorted than the optimized simulation did.

### Speech material experiment

**Fig. 4:**
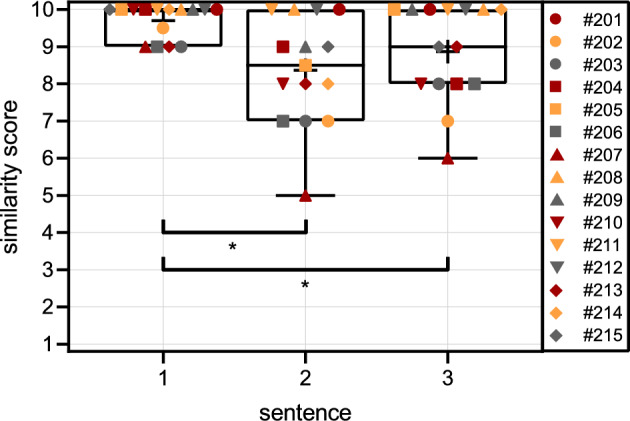
Similarity scores (1: no similarity to the CI sound, 10: signals are identical) of the 15 study participants for three sentences. The symbols of the individual study participants are the same as those in (Fig. 3). The boxes show the lower and upper quartiles (25 and 75th percentiles). The horizontal lines mark the median, and the crosses mark the means of the similarity scores across all the study participants. Whiskers indicate the minimum similarity score. *Indicates statistically significant differences between similarity scores of different sentences (*p* < 0.05, Bonferroni-corrected, Friedman ANOVA followed by sign tests).

The parameters of the optimized simulations were then applied to two additional sentences, and the participants were asked to evaluate them with a similarity score. The similarity scores of the three sentences are shown in (Fig. 4). Each symbol represents the similarity score assigned by one participant. On average, the second sentence was given a similarity score of 8.4 ± 1.5, and the third sentence was given a similarity score of 8.9 ± 1.3. We found significant differences among the similarity scores of the three sentences (*χ*^*2*^(2) = 16.595, *p* < 0.001). The sign tests showed a significant difference between the similarity scores of sentences 1 and 2 (*Z* = 3.015, *p* = 0.003) and sentences 1 and 3 (*Z* = 2.268, *p* = 0.048). The difference between the similarity scores of sentences 2 and 3 was not statistically significant (*Z* = 7.000, *p* = 0.21).

When the similarity scores are considered individually for each participant (see Fig. 4), we see that there are participants who rated each sentence with the same score (e.g., #208) and participants who rated the similarity differently for each sentence (e.g., #207). No participant rated the unfamiliar sentences higher than the first sentence.

## Discussion

### Screening experiment

One aim of this work was to create German language simulations for sound impressions with Nucleus CIs in CI users who had completed follow-up therapy (CI experience of more than two years). In addition to identifying the best-fitting modification technique, the screening experiment provided the experimenter with information on the sound impressions of the individual study participants. Good communication with the participants appeared critical to the success of this research. During the first experiment, the participants had the opportunity to identify certain sound aspects that were similar to their CI and to communicate this to the experimenter. Figure 1 shows that simple filters (low, high, and band-pass filters) best approximated the sound of the CI for the average patient. However, the scatter of the individual data was also the largest for these types of signal modifications.

The FFT-vocoder with a sine carrier appears to be a good match to the CI sound. This may be because the FFT vocoder mimics the n-of-m coding strategy^34,35^ used by the CI. Dorman et al. reported a median similarity score of 2.9 for sine vocoded speech and 1.9 for noise vocoded speech (scale: 0 to 10)^14^. These results are consistent with those of our study, as the sine FFT vocoder achieved a higher median similarity score than did the noise FFT vocoder. Peters et al. reported a similarity score of 6.8 (scale: 1 to 10) for vocoded speech^10^. The variations in median/mean similarity scores among the studies by Dorman et al., Peters et al. and our study may be due to differences in data analysis (e.g.,^10^: mean values over the best matches, present study: median for a fixed parameter set) and the programming of the vocoders^10,14^.

### Optimization experiment

The simulations created in the optimization experiment showed that the sound impressions of CI users varied even after completing the follow-up therapy. This variability in sound perception is in accordance with the results reported by Dorman et al.^15^. Thus, one CI sound does not exist, but several CI sounds exist. The variations in CI sounds in this study cohort can be caused, for example, by differences in CI settings (such as T and C levels), insertion depths, duration of deafness, time of usage or implant types. However, even in the small study population (n = 15), certain sound characteristics appeared to be preferable. For example, participants #201 and #214 required a band-pass filter with the same filter parameters to optimize their simulation. Participant #215 also required only a band-pass filter, which limited the frequencies more than participants #201 and #214. Participants #202 and #209 also had similar sound impressions with their CIs. Participants #205 and #212 required no changes to the original signal. This demonstrates, on the one hand, that a CI’s sound can subjectively match that of a normally hearing ear very closely and, on the other hand, that the sound perceptions of CIs can often be similar or even indistinguishable between individuals.

The variability in CI sounds between the studies of Dorman et al.^15^ and this study might be due to differences in hardware and software of the CI systems, device programming and patient-related factors (e.g., etiology and duration of deafness) of the investigated study participants. In this study, participants were aided with Nucleus devices, and in the study by Dorman et al.^15^, participants used MED-EL devices (Innsbruck, Austria). Different implant designs (e.g., number of electrode contacts and coding strategies) may lead to different sound impressions.

When the simulations are sorted according to the participants’ CI experiences, an experienced listener can perceive an adaptation of speech sound impressions toward the sound of a normal hearing ear. The four study participants who had the longest CI experience in this study (CI experience > 6 years) required no or very small changes. These results may indicate the relevance of neuronal plasticity, adaptation, and learning in the context of CI sound perception.

Notably, even the participants who required no or very small changes in CI simulations had no complete (100%) speech recognition in the speech recognition test. This was possible because the CI simulations presented in this study attempted to simulate CI sounds, not the functionality and processing mechanisms in CIs and CI users. Furthermore, previous studies have reported only a moderate correlation between sound quality and speech recognition^40,41^, indicating that sound quality and speech recognition are distinct measurements that should be considered separately. This observation became particularly evident during the study and prompted us to ask participants about it. All the participants confidently confirmed that they understood the study tasks correctly. However, they were unable to provide a definitive explanation. Some participants noted that hearing with a CI felt different and that more concentration was required for them to understand.

On average, a similarity score of 9.7 ± 0.5 was achieved for the optimized simulation. The optimization procedure led to a significant improvement in similarity compared with similarity scores in the screening experiment. Ten of the study participants indicated that their optimized simulations sounded exactly like their CI. Therefore, the CI simulations generated in this study appear suitable for counseling patients and their families, particularly in preoperative settings. For parents of pediatric CI candidates and SSD CI candidates, these simulations can help increase acceptance by illustrating the range of possible CI sound perceptions. This approach may help manage expectations and facilitate informed decision-making during counseling sessions.

However, the use of CI simulations for educational purposes should always be done with caution, and CI simulations should not be used in the sense of a “promise”. As discussed above, the coding strategy and other technical features might significantly influence the sound of a CI, and one CI sound does not exist. Therefore, a wide range of representative simulations, e.g., simulations developed for other CI manufacturers, are important to use them for educational purposes. Furthermore, the simulations presented in this study only offer a realistic assessment of the CI sounds of SSD CI users. Other effects associated with cochlear implantation (e.g., changes in quality of life) are not simulated^2,42^ . The sound perception of bilateral CI users might deviate because of variations in learning mechanisms affected by the contralateral ear.

The screening experiment led to a strong commitment of the study participants to the question of how their CI sounds. In this way, the participants were already presented with different sound aspects so that they were supported in describing their CI sounds in addition to the adjective list. Since there were already extensive discussions between the study participants and the experimenter during the first study task, this may have led to an improvement in the optimization process. Same time, participants had already heard the unprocessed speech signal more frequently through the first study task. Because this task also presented simulations that were only slightly altered to the normal-hearing ear (e.g., simulations 1–3, Table 1), an adaptation or training effect might have begun. This may have simplified and improved the optimization process, resulting in a better similarity score. Since the study participants were presented with ten different sentences in the screening experiment (and not only the sentence that was used for optimization), the influence of this effect is probably rather small. However, the mean similarity score in this study (9.7 ± 0.5) is comparable to that reported by Dorman et al. (8.8 ± 0.9,^15^).

In addition to low-pass filters, comb filters were used most often to optimize the simulations (4 study participants). Even if the filter bank in CIs is arranged logarithmically, the comb filters with a linear frequency distribution reflect the sound aspects of CI transmitted signals in some participants. To improve CI sounds toward more natural hearing for these patients, future research could focus on enhancing the filter bank implemented in the ACE strategy. To draw further conclusions regarding the optimization of CI technology, future studies should investigate CI simulations using different electrode array lengths, numbers and spacing of electrode contacts, as well as various coding strategies. The study design and the software sound tool used in the presented study provide a suitable framework for such investigations. Comparing the resulting sound impressions could help identify technical parameters that contribute to a more natural hearing experience and may guide future developments in CI design.

### Speech material experiment

In addition, we examined whether the parameters for signal changes to describe the CI sound are also an accurate approximation of the CI sound when applied to two further sentences with the same male speaker. When the parameters of the optimized simulation were applied to the two other sentences, the similarity scores were, on average, worse than those of the optimized sentences. However, the mean values of the similarity scores 8.4 ± 1.5 and 8.9 ± 1.3 still showed a close similarity to the CI sound (compare to the mean similarity score in^15^: 8.8).

For some participants, the similarity score did not depend on the speech material used (#201, #208, #211, #212). Thus, the simulation parameters may also be applied at least to other sentences of the OlKiSa with a male speaker. For the other study participants, a dependency on the speech material was observed. The fact that the similarity changes depending on the speech material may be due to the frequency dependency of the simulation parameters since the sentences have different frequency spectra. However, this might also be due to a training effect.

### Limitations of the study

A limitation of this study is that only the influence of words on CI similarity was investigated. The same speaker and the same language (German) were used throughout this study. The influence of the individual speaker, the biological sex of the speaker and language (speech rhythm, melody) on the similarity to the CI sound was not investigated in this study. Pseudo-sentences were not used, because real speech material has a meaning and context, making the simulations easier to present to patients in everyday clinical practice. Furthermore, whether the simulation parameters can be used to simulate the music perception of CI users cannot be determined on the basis of this study.

Due to the small sample size (n = 15), this was a purely exploratory study. Sound perception is very subjective. For that reason, there is no objective way to measure the sound of a CI, and we must rely on the subjective information provided by the participants. This individual optimization process hindered reproducibility, especially since participants may, as described by Dorman et al., focus on one particular acoustic aspect, thereby reducing reliability^15^. Subjective approaches such as the one applied in this study have inherent limitations, similar to speech audiometry, where a single examiner evaluates a patient’s responses. In this study, the evaluation was performed by an experimenter with in-depth knowledge of the underlying simulation algorithms, allowing for a structured and consistent interpretation of the participant’s descriptions. Notably, an age-dependent hearing threshold was used as the criterion for normal hearing. This means that some of the study participants had hearing loss of > 30 dB or high-frequency hearing loss, resulting in a (patho)physiological low-pass filter in their “normal hearing ear” to which the CI sound was compared.

Furthermore, usage time was not defined as an exclusion criterion and therefore varied among participants. For instance, participant #209 used the CI less frequently due to a recently developed comorbidity, whereas participant #205 had extensive CI experience with intensive all-day usage during the first years after implantation. Both participants achieved good results in the word recognition test with the CI and were thus included in the study.

## Conclusion

To summarize, this work makes the following three contributions to CI research. First, with the method adapted from Dorman et al.^15^, it was possible to create German CI simulations replicating the sound impressions patients have with Nucleus CIs. Second, we showed that despite all participants having a minimum of two years of CI experience, their sound impressions of their CIs varied significantly. The results suggest that with increasing CI experience, the perceived sound impressions may subjectively become closer to those of a normal hearing ear. Third, the parameters can also be applied to two further sentences spoken by the same speaker and still accurately describe the CI sound in most cases.

## Supplementary Information

Supplementary Information 1.

Supplementary Information 2.

Supplementary Information 3.

Supplementary Information 4.

Supplementary Information 5.

Supplementary Information 6.

Supplementary Information 7.

Supplementary Information 8.

Supplementary Information 9.

Supplementary Information 10.

Supplementary Information 11.

Supplementary Information 12.

Supplementary Information 13.

Supplementary Information 14.

Supplementary Information 15.

Supplementary Information 16.

Supplementary Information 17.

## Data Availability

All data generated or analyzed during this study are included in this published article and its supplementary information files.
