# Supplementary material for: Acoustic simulation of cochlear implant sound to approximate the perceptual experience of electric hearing
Source: Sci Rep. 2025 Nov 7;15:38997. doi: 10.1038/s41598-025-25711-z (PMC12595112; doi:10.1038/s41598-025-25711-z)
Supplement: Supplementary file 17 — Supplementary Information 17. [file 41598_2025_25711_MOESM17_ESM.docx]

| title | description |
| --- | --- |
| #201_sentence1.wav | Simulation optimized specifically for participant #201. |
| #202_sentence1.wav | Simulation optimized specifically for participant #202. |
| #203_sentence1.wav | Simulation optimized specifically for participant #203. |
| #204_sentence1.wav | Simulation optimized specifically for participant #204. |
| #205_sentence1.wav | Simulation optimized specifically for participant #205. |
| #206_sentence1.wav | Simulation optimized specifically for participant #206. |
| #207_sentence1.wav | Simulation optimized specifically for participant #207. |
| #208_sentence1.wav | Simulation optimized specifically for participant #208. |
| #209_sentence1.wav | Simulation optimized specifically for participant #209. |
| #210_sentence1.wav | Simulation optimized specifically for participant #210. |
| #211_sentence1.wav | Simulation optimized specifically for participant #211. |
| #212_sentence1.wav | Simulation optimized specifically for participant #212. |
| #213_sentence1.wav | Simulation optimized specifically for participant #213. |
| #214_sentence1.wav | Simulation optimized specifically for participant #214. |
| #215_sentence1.wav | Simulation optimized specifically for participant #215. |
| original_sentence1.wav | Original audio signal of sentence 1. |
